# RDFIO: extending Semantic MediaWiki for interoperable biomedical data management

**DOI:** 10.1186/s13326-017-0136-y

**Published:** 2017-09-04

**Authors:** Samuel Lampa, Egon Willighagen, Pekka Kohonen, Ali King, Denny Vrandečić, Roland Grafström, Ola Spjuth

**Affiliations:** 1 Department of Pharmaceutical Biosciences, Uppsala University, Uppsala, SE-751 24 Sweden; 2 Department of Bioinformatics - BiGCaT, NUTRIM, Maastricht University, P.O. Box 616, UNS50 Box 19, Maastricht, NL-6200 MD The Netherlands; 3 Institute of Environmental Medicine, Karolinska Institutet, Stockholm, SE-171 77 Sweden; 4 Division of Toxicology, Misvik Biology Oy, Turku, Finland; 5 FanDuel Inc, Edinburgh, UK; 6 Google Inc., 345 Spear Street, San Francisco, USA

**Keywords:** Semantic MediaWiki, MediaWiki, Wiki, Semantic Web, RDF, SPARQL, Wikidata

## Abstract

**Background:**

Biological sciences are characterised not only by an increasing amount but also the extreme complexity of its data. This stresses the need for efficient ways of integrating these data in a coherent description of biological systems. In many cases, biological data needs organization before integration. This is not seldom a collaborative effort, and it is thus important that tools for data integration support a collaborative way of working. Wiki systems with support for structured semantic data authoring, such as Semantic MediaWiki, provide a powerful solution for collaborative editing of data combined with machine-readability, so that data can be handled in an automated fashion in any downstream analyses. Semantic MediaWiki lacks a built-in data import function though, which hinders efficient round-tripping of data between interoperable Semantic Web formats such as RDF and the internal wiki format.

**Results:**

To solve this deficiency, the RDFIO suite of tools is presented, which supports importing of RDF data into Semantic MediaWiki, with metadata needed to export it again in the same RDF format, or ontology. Additionally, the new functionality enables mash-ups of automated data imports combined with manually created data presentations. The application of the suite of tools is demonstrated by importing drug discovery related data about rare diseases from Orphanet and acid dissociation constants from Wikidata. The RDFIO suite of tools is freely available for download via pharmb.io/project/rdfio.

**Conclusions:**

Through a set of biomedical demonstrators, it is demonstrated how the new functionality enables a number of usage scenarios where the interoperability of SMW and the wider Semantic Web is leveraged for biomedical data sets, to create an easy to use and flexible platform for exploring and working with biomedical data.

## Background

While much attention has been paid to the ever growing volumes of biological data from recently emerging high throughput technologies [1, 2], the biological sciences are importantly also characterised by the extreme complexity of its data. This complexity stems both from the incredible inherent complexity of biological systems, as well as from the vast number of data formats and assisting technologies developed by the scientific community to describe these systems. In order to provide a coherent description of biological systems making use of the data sources available, data integration is of central importance [3]. Also, while there are vast amounts of biological data publicly available, for many problems the necessary data to be integrated is still comparably small, however complex, and in need of organization before integration.

Biological data integration is an active field of research and a number of strategies have been presented for addressing the data integration problem [4, 5]. Data integration involves a wide range of considerations, including data governance, data licensing issues and technology. In terms of technical solutions, the most central solution for data integration proposed so far is a set of flexible and interoperable data formats and technologies commonly referred to as the “Semantic Web” [6, 7], with its main underlying data format and technology, the “Resource Description Framework” (RDF) [8, 9], accompanied by technologies such as the SPARQL Protocol and RDF Query Language (SPARQL) [10] and the Web Ontology Language (OWL) [11].

The power of these data formats and technologies lie in their ability to capture data, ontologies and linking information between multiple ontologies in a single underlying serialisation format. This enables disparate user communities to create data sets adhering to different ontologies and adding linking information between datasets afterwards. It furthermore enables generic tools to leverage the ontology and linking information to present data from multiple sources in a coherent, integrated fashion, on-demand.

While most biological data today is not available in RDF format, initiatives such as the Bio2RDF project [12] are tackling this by providing a way to convert publicly available datasets in non-RDF formats to RDF, by writing so called *rdfizers* for each dataset, and using a URI normalisation scheme developed as part of the project to ensure that URIs referring to the same object are encoded in the same way [12]. More recent examples of well supported RDF-ization efforts of biological data are the Open PHACTS project and platform [13, 14], providing an integrated environment for working with data and tools related to drug discovery, and the EBI RDF [15] platform, which provides data from multiple of EBI’s biological data sources in an integrated semantic data layer where connections between multiple data sources can easily be made, e.g. at the time of querying the data via the SPARQL endpoint made available.

The heterogeneous nature of biological data also means that the task of managing, annotating, curating and verifying it is prohibitively complex for a single researcher to carry out because of the knowledge needed to understand the many biological systems, data formats and experimental methods involved. This highlights the importance of effective collaborative tools in biology, to allow experts from multiple sub-fields within biology to work together to build integrated biological data sources. For example, in the chemicals and nanomaterials safety science field, semantically annotated databases with domain-specific ontologies are being used to standardise collaborative community data entry and curation [16, 17].

One successful approach to enable flexible collaboration on biological data is wiki systems [18, 19]. Wikis facilitate collaboration by removing technological complexity from the editing process, allowing anyone with access to the wiki to edit any part of it. Instead of complicated authentication controls, it generally manages trust in the content by saving every change in the system as a new revision, not allowing deletion of content, and logging which user did the change. This way, other users can review changes made and make any corrections needed or simply roll back changes that do not fulfil the criteria set up for the data source, resulting in a simple and friendly environment for editing content for any user.

Plain-text wiki systems have a large drawback though: They only allow plain text to be stored while lacking support for structured, machine-readable, data. To solve this problem a solution proposed by a number of groups is to combine a wiki system with support for storing structured data in the form of semantic “facts”, consisting of a property–value pair, closely mapping to the predicate and object in RDF triples, and resulting in a combination of the ease-of-use, and flexibility of wikis, with the ability to create structured, machine-readable data. A review of numerous Semantic Wiki implementations is available in [20]. A recent wiki approach for databases was introduced with the Wikibase software used by the Wikidata project [21] and is already used in the life sciences [22, 23]

Semantic MediaWiki (SMW) [24] is currently one of the most known and widely used semantic wikis. One of the factors for its success is that it is based on MediaWiki [25], the software powering Wikipedia and thousands of other wikis. SMW allows to combine the unstructured content of typical MediaWiki wikis, with structural semantic content, encoded using a dedicated syntax that extends the MediaWiki syntax.

SMW has found a number of uses in biomedical contexts. Apart from often being used as an internal wiki system at many labs, it has also been used in publicly available resources, including MetaBase [26], a wiki-database of biological databases, SNPedia [27], a wiki-database focusing on medically and personally relevant Short Nucleotide Polymorphisms (SNPs), the Gene Wiki portal on Wikipedia [28], and a catalog of a transcriptome based cellular state information in mammalian genomes in the FANTOM5 project [29].

SMW has many features to make it interoperable with the rest of the Semantic Web, such as export of normal wiki pages and the “facts” that relate them, as RDF/XML, export of “Categories” as OWL classes and so called “Concepts” [30] as OWL class descriptions [31]. Also, integration with third party semantic data stores is possible via third party plugins. It also has a feature to enable so called “Vocabulary import”, which is a way to link properties in the wiki to predicates of external Semantic Web ontologies, by manually creating special articles that define these links [32].

A notable limitation of SMW is the lack of a general RDF data import function. That is, the ability to do automatic batch import of RDF datasets into the wiki. Note that such a functionality is distinct from the so called “vocabulary import” feature described earlier, which only enables manual linking of properties to ontology items, but no automatic import of data, and no support for importing plain RDF triples (OWL *individuals*), regardless of whether an ontology is used or not.

This lack of a general RDF import function means that usage scenarios such as bootstrapping new wikis from existing data sources, or round-tripping between the SMW data structure and the RDF data format used in the wider Semantic Web, are not possible without external tools. This has important consequences, since for example round-tripping between SMW and RDF could provide important benefits for data integration. As already mentioned, wiki systems have proven to be excellent platforms for collaborative editing. Thus, by storing RDF data in a text format closely resembling normal wiki syntax, it is possible to leverage the benefits of a proven wiki platform to lower the barrier to entry for new users to start editing semantic data. In other words, allowing full round-trip between SMW and RDF data sets would allow to present RDF data in a format more apt to collaborative editing and curation, after which it can be exported again into the RDF format for use in the wider Semantic Web.

Additionally, import of RDF data sets into SMW would allow creating mash-ups, combining automatically imported data sets of moderately large size with manually created presentations of this data using the querying and visualisation tools available in SMW or its eco-system of third-party libraries. Based on these possibilities it can be concluded that RDF import in SMW is an enabler of a number of usage scenarios useful in data integration, including making working with semantic data easier for users without deep knowledge of the Semantic Web.

There exist a few solutions for semantic data import in SMW, developed as third-party extensions. Among these, Fresnel Forms [33] is focused on the import of an ontology structure rather than plain RDF triples (OWL *individuals*), and also requires running the Protégé software outside of the wiki installation. Furthermore, the Linked Wiki Extension [34] allows import of plain RDF triples but does this by importing the triples into an external triple store rather than inserting the data as SMW “facts” inside the wiki source text, which is required for being able to further modify the data in the wiki format.

To solve this lack of plain triples RDF data import into SMW facts in the wiki text, a set of tools and SMW extensions commonly named as the “RDFIO suite” was developed. These tools and extensions are presented below together with biomedical demonstrators of the benefits of the methodology.

## Implementation

The RDFIO suite consists of the following parts:

1. A web form for importing RDF data via manual entry or copy-and-paste.
2. A SPARQL endpoint allowing both querying and creation of RDF triples via an INSERT INTO statement, as well as RDF export by running CONSTRUCT queries.
3. A SPARQL endpoint replicator, which can import semantic data from an external SPARQL endpoint (in essence creating a mirror of the data set).
4. A command-line import script for import of RDF data stored in a file.
5. A command-line export script for export for RDF data into a file.
6. A standalone command-line tool for converting RDF triples into a MediaWiki XML file, for further import using MediaWiki’s built-in XML import function, named rdf2smw (referred to as rdf2smw below).

**Fig. 1:**
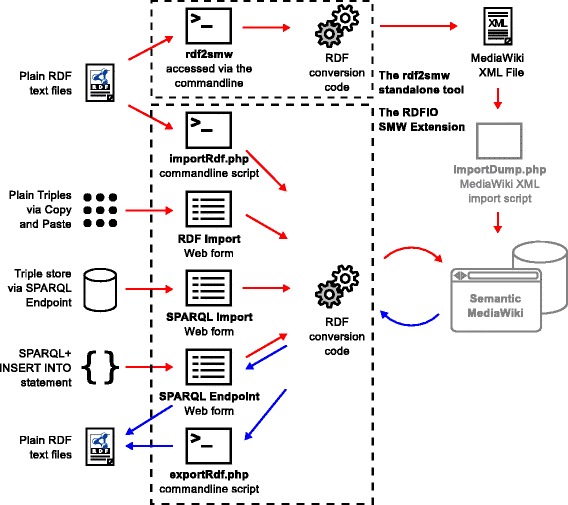
Overview of the intended usage for the different parts of the RDFIO suite. The figure shows how RDF data can be retrieved from a set of different sources, as well as being exported again. The parts belonging to the RDFIO SMW extension and the rdf2smw tool are marked with dashed lines. The newly developed functionality in this paper is drawn in black while already existing functionality in MW and SMW is drawn in grey color. Red arrows indicate data going into (being imported into) the wiki, while blue arrows indicate data going out of (being exported from) the wiki. From top left, the figure shows: i) how RDF data files can be batch imported into SMW either by using the rdf2smw tool to convert them to MediaWiki XML for further import using MediaWiki’s built-in XML import function, or via the importRdf.php commandline script in the RDFIO SMW extension, ii) how plain triples (OWL *individuals*) can be imported from text files, or from web pages via copy and paste into a web form, iii) how a remote triple store exposed via a SPARQL endpoint can be replicated by entering the SPARQL endpoint URL in a web form, iv) how new RDF data can be created manually or dynamically in the SPARQL endpoint via SPARQL INSERT INTO statements supported by the SPARQL+ extension [44] in the ARC2 library, and finally, v) how data can also be exported via the SPARQL endpoint, using CONSTRUCT queries, or vi) by using the dedicated exportRdf.php commandline script

Tools 1-5 above were developed in the PHP programming language, as modules of a common MediaWiki extension called RDFIO. An overview picture of how these parts are related to each other is available in Fig. 1. Tool 6 above, which is a standalone tool, was developed in the Go programming language to provide shorter execution times for the RDF-to-wiki page conversion of large data sets.

Tools 1-3 are implemented as MediaWiki Special-pages, each providing a page with a web form related to their task. Tools 1-5 all rely on the PHP based RDF library ARC2 [35]. ARC2 provides its own MySQL-based data store which is used for all its functions and which is installed in the same database as the MediaWiki installation when installing RDFIO. To enable the ARC2 data store to capture the data written as facts in the wiki a custom SMW data store was developed. It hooks into each page write and converts the SMW facts of the page into the RDF format used in the ARC2 store.

The most resource demanding part of the import process is the creation of wiki pages in the MediaWiki software. Thus, to enable previewing the structure of the wiki pages, most importantly the wiki page titles chosen, before running the actual import, the standalone tool in 6 above was developed. By generating a MediaWiki XML file as an intermediate step before the import, the user has the option to view the wiki page content and titles in the MediaWiki XML file in a text editor before running the file through MediaWiki’s built-in import function. While this is not a mandatory step, it can be useful for quickly identifying whether any configuration settings should be changed to get more useful wiki page titles, before the more time-consuming MediaWiki import step is initiated.

The limitation of using the standalone tools is that any manual changes would be overwritten by re-running the import (although an old revision with the manual change will be kept, like always in MediaWiki). We thus anticipate that the external tool will only be used for the initial bootstrapping of the wiki content, while any imports done after manual changes have been made, will be done using the PHP based import tool mentioned above, which supports updating facts in place without overwriting manual changes.

## Results and discussion

To solve the lack of RDF import in SMW, the RDFIO suite was developed, including the RDFIO SMW extension and the standalone rdf2smw tool. The SMW extension consists of a set of functional modules, each consisting of a MediaWiki Special page with a web form, or a commandline script. A description of the features and intended use of each of these parts follows. See also Fig. 1 for a graphical overview of how the different parts fit together.

### RDF import web form

The RDF import web form allows the user to import RDF data in Turtle format either from a publicly accessible URL on the internet, by manually entering or copy-and-pasting the data into a web form. This allows users to import small to moderate amounts of RDF data without the need for command-line access to the computer where the wiki is stored, as is often required for batch import operations. The drawback of this method is that since the import operation is run as part of the web server process, it is not suited for large amounts of data. This is because it would then risk using up too much computational resources from the web server and making the website unresponsive for other users for a single-server setting, which is often used in the biomedical domain.

### SPARQL import web form

The SPARQL import web form allows importing all data from an external triple store exposed by a publicly accessible SPARQL endpoint. Based on an URL pointing to an endpoint it will in principle create a mirror of it, since the data imported into the wiki will in turn be exposed as a SPARQL endpoint (see the corresponding section below). The import is done with a query that matches all triples in the external triple store (In technical terms, a SPARQL clause of the form: WHERE { ?s ?p ?o }). In order not to put too much load on the web server, the number of triples imported per execution is by default limited by a pre-configured limit. This enables performing the import in multiple batches. The user can manually control the limit and offset values, but the offset value will also be automatically increased after each import, so that the user can simply click the import button multiple times, to import a number of batches with the selected limit of triples per batch.

### SPARQL endpoint

**Fig. 2:**
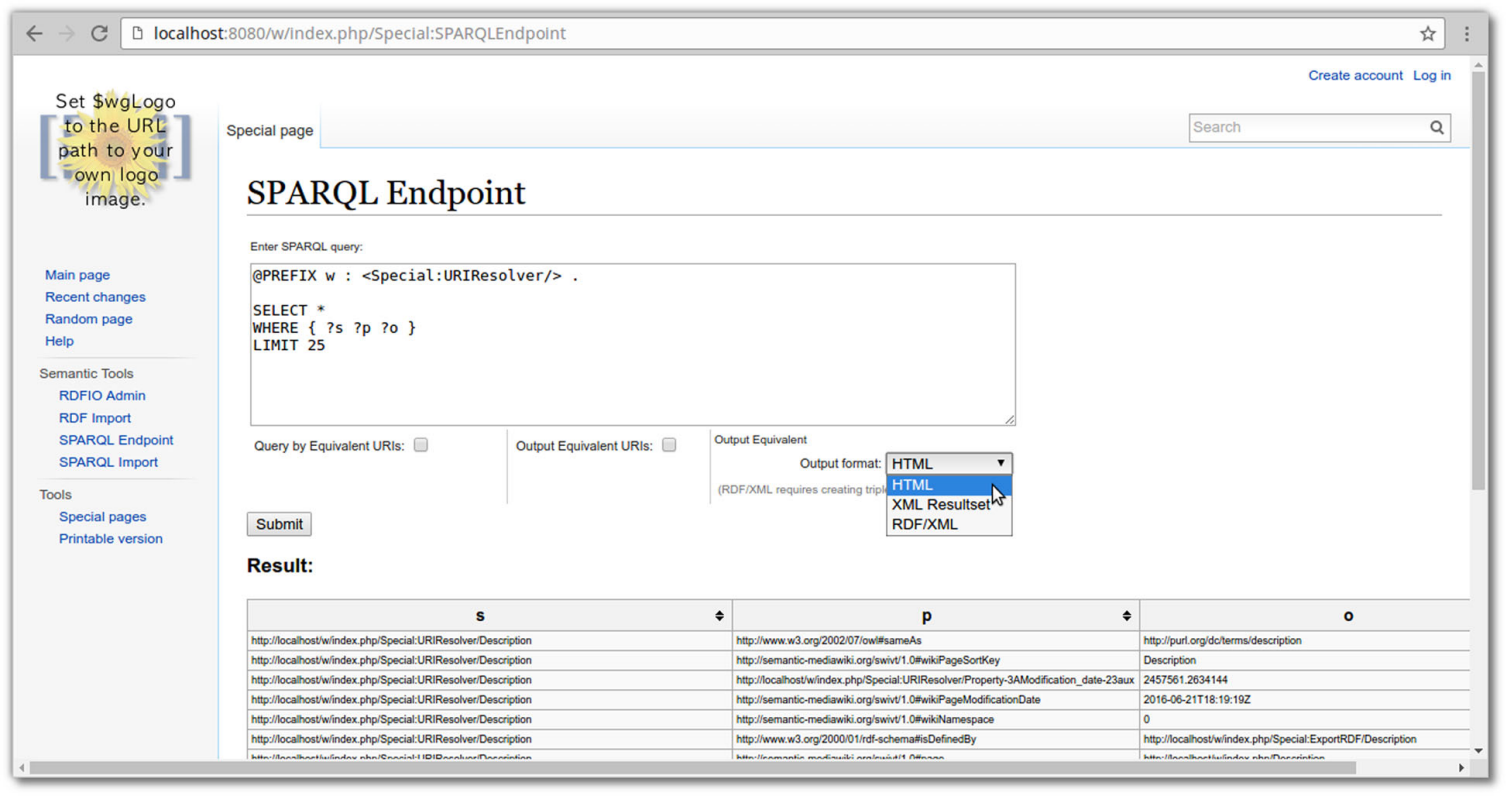
A screenshot of the SPARQL endpoint web form in RDFIO. A key feature of the SPARQL endpoint is the ability to output the original RDF resource URIs of wiki pages, that were used in the original data imported. This can be seen by the checkbox option named “Query by Equivalent URIs” and “Output Equivalent URIs”, named so because the original URIs are stored using the “Equivalent URI” special property, on each page created in the import

The SPARQL endpoint (see Fig. 2) exposes all the semantic data in the wiki as a web form where the data can be queried using the SPARQL query language. The endpoint also allows external services to query it via the GET or POST protocols. It can output either a formatted HTML table for quick previews and debugging of queries, a machine-readable XML result set, or full RDF triples in RDF/XML format. The RDF/XML format requires the use of the CONSTRUCT keyword in the SPARQL query to define the RDF structure to use for the output. Using CONSTRUCT to output RDF/XML basically amounts to a web based RDF export feature, which is why a separate RDF export web form was not deemed necessary.

The SPARQL endpoint also allows adding new data to the wiki using the INSERT INTO statement available in the SPARQL+ extension supported by ARC2.

### RDF import batch script

**Fig. 3:**
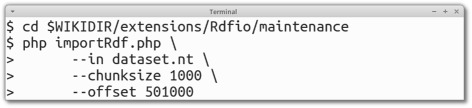
Usage of the command-line import tool in RDFIO. The figure shows examples of shell commands to use to import an RDF dataset, in this case in N-triples format, saved in a file named dataset.nt. The steps are: i) Change directory into the RDFIO/maintenance folder, and then ii) execute the importRdf.php script. One can set the variables --chunksize to determine how many triples will be imported at a time, and --offset to determine how many triples to skip in the beginning of the file, which can be useful if restarting an interrupted import session. The $WIKIDIR variable represents the MediaWiki base folder

The batch RDF import batch script (importRdf.php) is executed on the command-line, and allows robust import of large data sets. By being executed using the standalone PHP or HHVM (PHP virtual machine) [36, 37] executable and not the web server process, it will not interfere with the web server process as much as the web form based import. It will also not run into the various execution time limits that are configured for the PHP process or the web server. While a batch-import could also be implemented using the web form by using a page reload feature, or an AJAX-based JavaScript solution, this is a more complex solution that has not yet been addressed due to time constraints. Executing the batch RDF import script in the terminal can look like in Fig. 3.

### Stand-alone RDF-to-MediaWiki-XML conversion tool (rdf2smw)

The rdf2smw tool uses the same strategy for conversion from RDF data to a wiki page structure as the RDFIO extension but differs in the following way: Whereas the RDFIO extension converts RDF to wiki pages and writes these pages to the wiki database in one go, the standalone tool first converts the full RDF dataset to a wiki page structure and writes it to an XML file in MediaWiki’s XML import format, as illustrated in Fig. 1. This format is very straightforward, storing the wiki page data as plain text, which allows to manually inspect the file before importing it.

Programs written in Go are generally orders of magnitude faster than similar programs written in PHP. This performance difference together with the fact that the execution of the standalone rdf2smw tool is separate from the web server running the wiki is crucial when importing large data sets (consisting of more than a few hundred triples) since the import requires demanding data operations in memory such as sorting and aggregation of triples per subjects. This is the main reason why this external tool was developed.

**Fig. 4:**
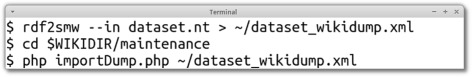
Command-line usage of the rdf2smw tool. The figure shows the intended usage of the rdf2smw command line tool. The steps are, one per line in the code example: i) Execute the rdf2smw tool to convert the RDF data into a MediaWiki XML file. ii) Change directory into the MediaWiki maintenance folder. iii) Execute the importDump.php script, with the newly created MediaWiki XML file as first argument. The $WIKIDIR variable represents the MediaWiki base folder

The usage of the tool together with MediaWiki’s built-in XML import script is illustrated in Fig. 4.

### RDF export batch script

The RDF export batch script (exportRdf.php) is a complement to the RDF export functionality available in the SPARQL endpoint, which analogously to the import batch script allows robust export of large data sets without the risk for time-outs and other interruptions that might happen to the web server process or the user’s web browser.

**Fig. 5:**
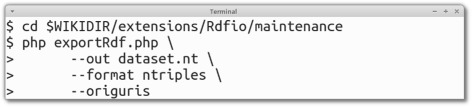
Usage of the command-line export tool in RDFIO. The figure shows examples of shell commands to use to export an RDF dataset, in this case in N-triples format, into a file named dataset.nt. The steps are: i) Change directory into the RDFIO/maintenance folder, and then ii) execute the exportRdf.php script, selecting the export format using the --format parameter. The --origuris flag tells RDFIO to convert SMW’s internal URI format back to the URIs used when originally importing the data, using the linking information added via SMW’s “Equivalent URI” property

Executing the batch RDF export script in the terminal can look like in Fig. 5.

### An overview of the RDF import process

As can be seen in Fig. 1, all of the import functions run through the same RDF-to-wiki conversion code except for the rdf2smw tool which has a separate implementation of roughly the same logic in the Go programming language.

**Fig. 6:**
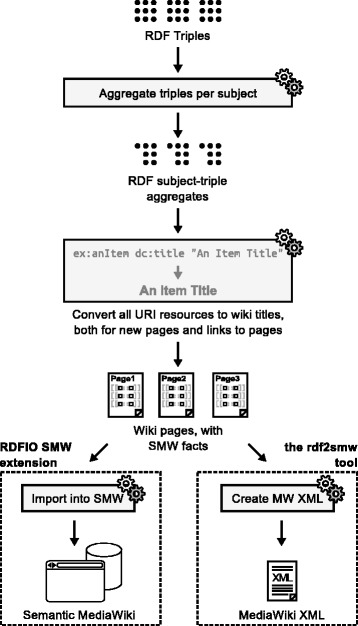
A simplified overview of the RDF to wiki page conversion process. The figure shows in a somewhat simplified manner, the process used to convert from RDF data to a wiki page structure. Code components are drawn as grey boxes with cog wheels in the right top corner, while data are drawn as icons without a surrounding box. From top to bottom, the figure shows how RDF triples are first aggregated per subject, then converted into one wiki page per subject, while converting all URIs to wiki titles, for new pages and links to pages, where-after the pages are either written directly to the wiki database (the RDFIO SMW extension), or converted to XML and written to files (the standalone rdf2smw tool)

The process is illustrated in some detail in Fig. 6 and can be briefly be described with the following processing steps:

- All triples in the imported chunk (number of triples per chunk can be configured for the commandline import script while the web form imports a single chunk) are aggregated per subject resource. This is done since each subject resource will be turned into a wiki page where predicate-object pairs will be added as SMW fact statements consisting of a corresponding property-value pair.
- WikiPage objects are created for each subject resource. The title for this page is determined from the Uniform Resource Identifier (URI) of the subject, or from some of the predicates linked to this subject, according to a scheme described in more detail below.
- All triples with the same subject, which have now been aggregated together, are turned into SMW facts (property-value pairs), to be added to the wiki page. Predicate and object URIs are converted into wiki page titles in the process, so that the corresponding property and value will be pointing to valid wiki page names. Naturally, if the object is a literal rather than an URI, no transformation will be done to it. During this process the pages corresponding to the created property titles are also annotated with SMW data type information, based on XML Schema type information in the RDF source data.
- Optionally, the facts can be converted into a MediaWiki template call, if there is a template available that will write the corresponding fact, by the use of its parameter values.
- In the rdf2smw tool only, the wiki page content is then wrapped in MediaWiki XML containing meta data about the page, such as title and creation date.
- In the RDFIO SMW extension only, the wiki page objects are now written to the MediaWiki database.

### Converting URIs to user friendly wiki page titles

The primary challenge in the described process is to figure out user friendly wiki titles for the resources represented by URIs in the RDF data. This is done by trying out a defined set of strategies, stopping as soon as a title could be determined. The strategies start with checking if there is already a page available connected to the URI via an *Equivalent URI* fact in the wiki text. If this is the case, this existing title (and page) will be used for this triple. If that is not the case, the following strategies are tried in the stated order: 1) If there are any properties commonly used to provide a title or label for a resource, such as dc:title from the Dublin Core ontology [38], the value of that property is used. 2) If a title is still not found, the base part, or “namespace” of the URI is shortened according to an abbreviation scheme provided in the RDF dataset in the form of namespace abbreviations. 3) Finally, if none of the above strategies could provide an accepted title, the “local part” of the URI (The part after the last / or # character in the URL) is used.

### Performance

Table 1 provides information about the time needed to import a given number of triples (100, 1000, 10000 or 100000) drawn as subsets from a test dataset (the Comparative Toxicogenomics Database [39], converted to RDF by the Bio2RDF project), using the RDF SMW extension directly via the importRdf.php commandline script, as well as by alternatively converting the data to MediaWiki XML files with the rdf2smw tool and then importing them using MediaWiki’s importDump.php script. Note that when importing using the rdf2smw tool the import is thus performed in two phases.

**Table 1:** Execution times for importing RDF data into SMW using the importRdf.php script in the RDFIO extension (column 2) and converting to MediaWiki XML files using the rdf2smw tool and then importing the generated XML files with MediaWiki’s built-in XML import tool respectively (column 3 and 4), for a few different dataset sizes (column 1)

Number of	Import RDF	Convert to XML	Import XML
Triples	(RDFIO extension)	(rdf2smw tool)	(MediaWiki XML import)
100	24 s	0.00 s	17 s
1000	179 s (2m59s)	0.02 s	81 s (1m21s)
10000	1652 s (27m32s)	0.3 s	683 s (11m23s)
100000	16627 s (4h37m7s)	18 s	7063 s (1h57m43s)

The tests were performed in a VirtualBox virtual machine running Ubuntu 15.10 64bit, on a laptop running Ubuntu 16.04 64bit. The laptop used was a 2013 Lenovo Thinkpad Yoga 12 with a 2-core Intel i5-4210U CPU, with base and max clock frequencies of 1.7 GHz and 2.7 GHz respectively, and with 8 GB of RAM. The PHP version used was PHP 5.6.11. Time is given in seconds and where applicable also in minutes and seconds, or hours, minutes and seconds.

Manual testing by the authors show that the performance of an SMW wiki is not noticeably affected by multiple users reading or browsing the wiki. An import process of many triples can temporarily slow down the browsing performance for other users because of table locking in the database, though. This is a characteristic common to MediaWiki wikis, when a large import operation is in progress, or if multiple article updates are done at the same time, unless special measures are taken, such as having separate, replicated, database instances for reading, to alleviate the load on the primary database instance.

### Continuous integration and testing

The fact that RDFIO is an extension to a larger software (SMW), which itself is an extension of MediaWiki and that much of their functionality depends on state in a relational database, has added complexity to the testing process. Recently though, continuous integration systems as well as improved test tooling for MediaWiki and SMW has enabled better automated testing also for RDFIO. We use CircleCI as continuous integration system and results from this and other services are added as indicator buttons on the README file on the respective GitHub repositories.

As part of the build process, system tests are run for the RDF import function and for the RDF export function, verifying that the exported content matches the data that was imported. In addition, work has been started to add unit tests. User experience testing has been carried out in real-world projects mentioned in the introduction, where some of the authors were involved [16, 17].

### Round-tripping

**Fig. 7:**
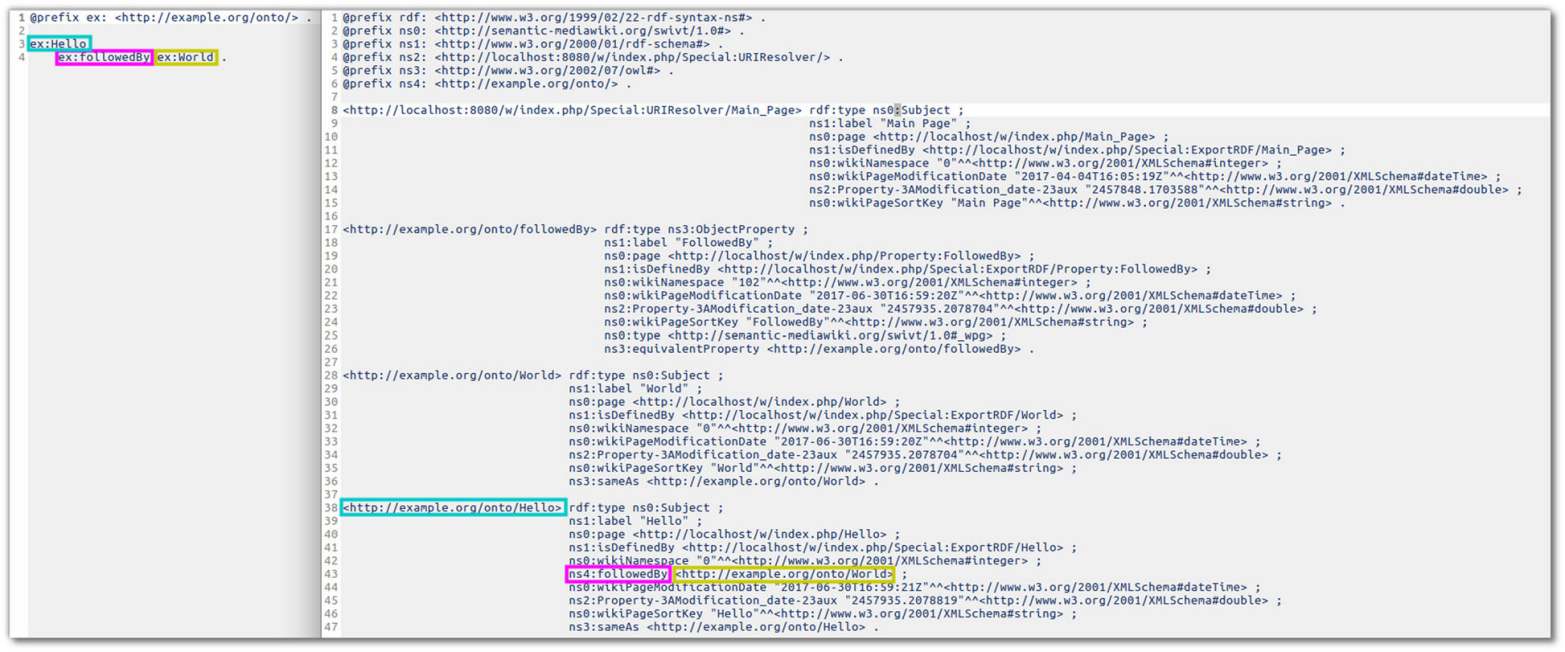
A comparison between data before and after an import/export round-trip. This figure shows to the left a dataset containing one single triple in turtle format. To the right is shown the data resulting from performing an import/export round-trip – that is, importing the initial data into a virtually blank wiki (The wiki front page “Main Page” being the only page in the wiki) and then running an export again. It can be seen in the exported data how i) The “Main Page” adds a certain amount of extra data, and ii) how there is a substantial amount of extra metadata about each resource added by SMW. The subject, predicate and value of the initial triple is color-coded with the same colours in both code examples (both before and after) to make it easier to find

As mentioned above, a system test for the round-tripping of data via the RDF and import and export functions is run, to ensure that no data is corrupted in the process. It is worth noting though that the RDF export will generally output more information than what is imported. This is because SMW does store certain meta data about all pages created, such as modification date etc. In the system test, these data are filtered out so that the test checks only consistency of the triples that were imported using RDFIO. An example of the difference between the imported and exported data can be seen in Fig. 7.

### Known limitations

At the time of writing this, we are aware of the following limitations in the RDFIO suite of tools:

- The rdf2smw tool supports only N-Triples format as input.
- There is currently no support for importing triples into separate named graphs, such that e.g. imported and manually added facts could be separated and exported separately.
- There is no functionality to detect triples for removal, if updating the wiki with a new version of a previously imported dataset, containing deprecated or having some triples simply removed.
- Cases with thousands of triples for a single subject leading to thousands of fact statements on a single wiki page – while technically possible – could lead to cumbersome manual editing.

These limitations are planned to be addressed in future versions of the tool suite.

### Demonstrators

#### Demonstrator I: Orphanet - rare diseases linked to genes

An important usage scenario for RDFIO is to visualise and enable easy navigation of RDF data by bootstrapping an SMW instance from an existing data source. To demonstrate this, the open part of the Orphanet dataset [40] was imported into SMW. Orphanet consists of data on rare disorders, including associated genes. The dataset was already available in RDF format through the Bio2RDF project [12], from where the dataset was accessed and imported into SMW. This dataset consisted of 29059 triples and was first converted to MediaWiki XML using the standalone rdf2smw tool, which was then imported using MediaWiki’s built-in XML import script. This presented an easy to use platform for navigating the Orphanet data, including creating listings of genes and disorders. Some of these listings are created automatically by SMW but additional listings can also be created on any page in the wiki, including on the wiki pages representing RDF resources, by using the template feature in MediaWiki in combination with the inline query language in SMW [41].

**Fig. 8:**
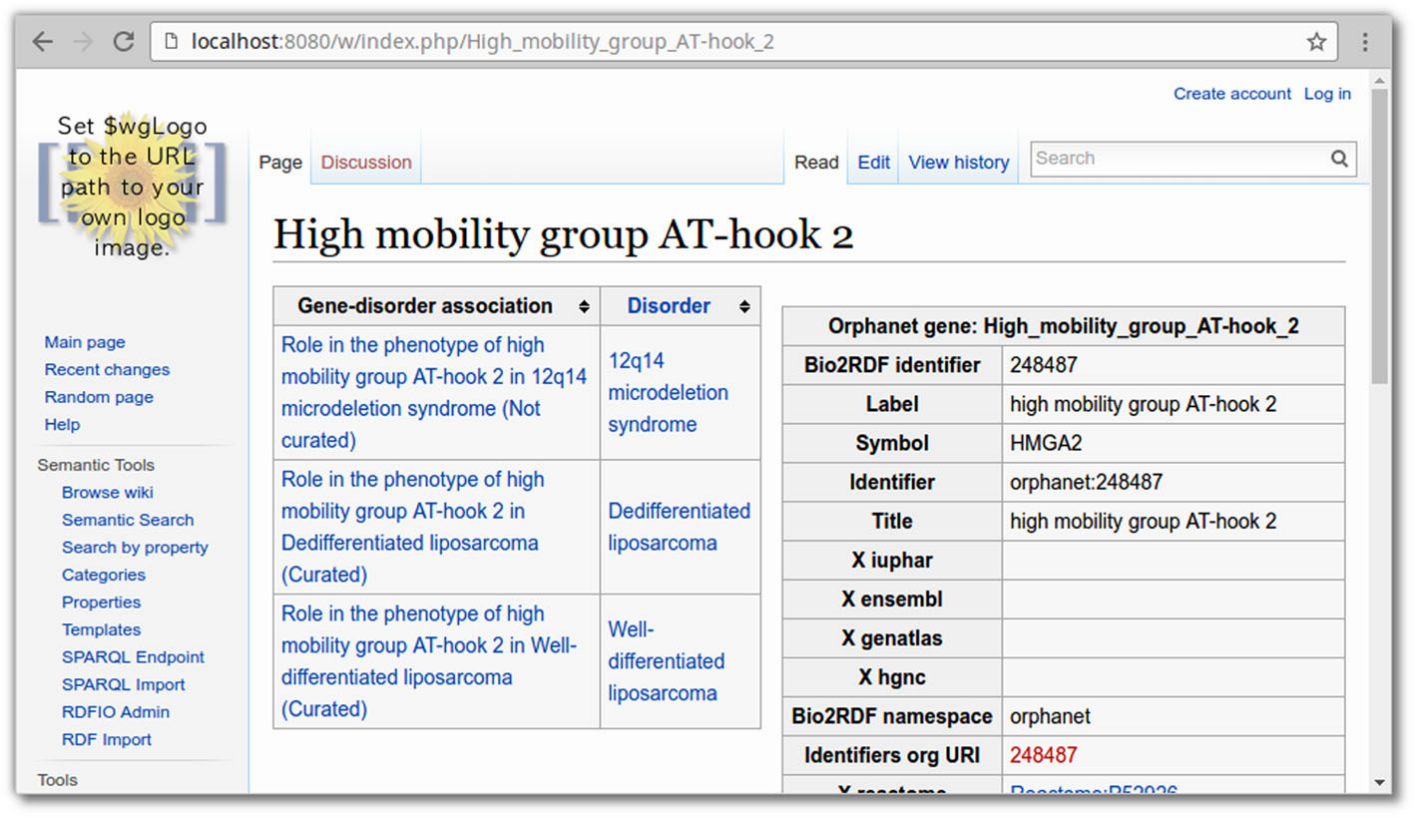
Screenshot of a wiki page for a gene in the Orphanet dataset. In the middle of the page, the listing of gene disorder associations and the corresponding disorders is shown. Note that these details are not entered on this page itself, but are queried using SMW’s inline query language and dynamically displayed. To the right are details entered directly on the page

An example of a useful user-created listing on an RDF node, was to create a listing of all the disorder-gene associations linking to a particular gene and the corresponding disorder, on the templates for the corresponding gene pages (For an example, see Fig. 8). In the same way, a listing of the disorder-gene association linking to particular disorders and the corresponding genes, was created on the templates for the corresponding disorder pages.

This example shows how it is possible, on a wiki page representing an RDF resource, to list not only information directly linked to this particular resource, but also information connected via intermediate linking nodes. Concretely, in the example shown in Fig. 8 we list a resource type (*diseases*) on a page representing a *gene* even though in the RDF data diseases are not *directly* linked to genes. Instead they are linked via an intermediate “gene-disorder association” node.

#### Demonstrator II: DrugMet - cheminformatics/metabolomics

The DrugMet dataset is an effort at collecting experimental pK _*a*_ values extracted from the literature, linked to the publication from which it was extracted, and to the chemical compounds for which it was measured. The DrugMet dataset was initially created by manually adding the details in a self-hosted Semantic MediaWiki. The data was later transferred to the Wikidata platform [21] for future-proofing and enabling access to the data for the wider community.

This demonstrator highlights how this data could be further curated by extracting the data again from Wikidata into a locally hosted SMW for further local curation.

The data was exported from Wikidata using its publicly available SPARQL REST interface [42]. The extraction was done using a CONSTRUCT query in SPARQL allowing to create a custom RDF format specifically designed for the demonstrator. For example, in addition to the publication and compound data, the query was modified to include rdf:type information for all the compounds, which is used by the RDFIO command line tool to generate a MediaWiki template call and corresponding template, for all items of this type.

**Fig. 9:**
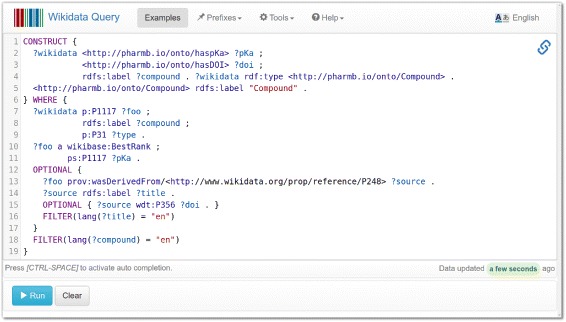
The SPARQL query for extracting DrugMet data. This screenshot shows the SPARQL query for extracting DrugMet data in Wikidata’s SPARQL endpoint web form. This query can be accessed in the Wikidata SPARQL endpoint via the URL: goo.gl/C4k4gx

**Fig. 10:**
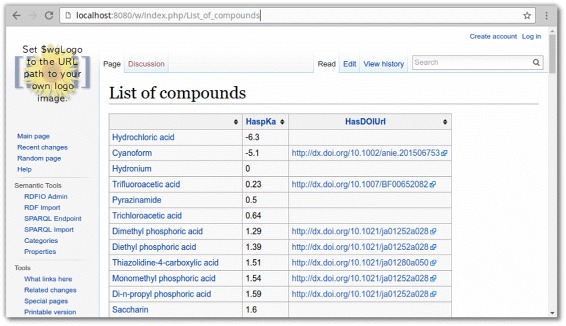
A dynamic listing of DrugMet data. The listing shows a locally hosted SMW wiki with a list of compounds and related information. The list is a custom, dynamically generated listing of Compound name, pK _*a*_ value and a link to the publication from which each pK _*a*_ value was extracted, created using SMW’s inline query language

After the data was imported into a local SMW wiki, it allowed to create a page with an SMW inline query displaying a dynamically sorted list of all the compounds, their respective pK _*a*_ values, and links to the publications from where the pK _*a*_ values were originally extracted. The query for this extraction is shown in Fig. 9, and the list is shown in Fig. 10.

#### Implications of the developed functionality

The demonstrators above show that the RDFIO suite of tools is successfully bridging the worlds of the easy-to-use wiki systems and the somewhat more technically demanding wider Semantic Web. This bridging has opened up a number of useful scenarios for working with semantic data in a flexible way, where existing data in semantic formats can easily and flexibly be combined by using the templating and querying features in SMW. This leads to a powerful experimentation platform for exploring and summarising biomedical data, which earlier was not readily accessible.

### Availability

- Complete information about the RDFIO project can be found at pharmb.io/project/rdfio
- A canonical location for information about the RDFIO SMW extension is available at MediaWiki.org at www.mediawiki.org/wiki/Extension:RDFIO
- All the software in the RDFIO suite is available for download on GitHub, under the RDFIO GitHub organisation, at github.com/rdfiowhere the RDFIO SMW extension is available at github.com/rdfio/rdfio, the rdf2smw tool at github.com/rdfio/rdf2smw and an automated setup of a virtual machine with a fully configured SMW wiki with RDFIO installed is available at github.com/rdfio/rdfio-vagrantbox.

### Outlook

Planned future developments include enhancing the rdf2smw tool with support for more RDF formats as input.

Further envisioned development areas are:

iv) Separating the ARC2 data store and SPARQL endpoint into a separate extension, so that the core RDFIO SMW extension does not depend on it. This could potentially improve performance of data import and querying, as well as make the core RDFIO extension easier to integrate with external triple stores via SMW’s triple store connector. v) Exposing the RDF import functionality as a module via MediaWiki’s action API [43]. This would allow external tools to talk to SMW via an established web interface. vi) Allowing to store domain specific queries tied to certain properties that can, on demand, pull in related data for entities of a certain ontology such as gene info from Wikidata, for genes.

## Availability and requirements

**Project name:** RDFIO

**Project home page:** https://pharmb.io/project/rdfio

**Operating system(s):** Platform-independent (Linux, Windows, Mac)

**Programming language:** PHP (The RDFIO SMW extention), Go (The rdf2smw tool)

**Other requirements:** A webserver (Apache or Nginx), A MySQL compatible database, MediaWiki, Semantic MediaWiki, ARC2 (RDF library)

**License:** GPL2 (The RDFIO SMW extention), MIT (The rdf2smw tool)

## Conclusions

The RDFIO suite of tools for importing RDF data into SMW and exporting it again in the same RDF format (expressed in the same ontology) has been presented. It has been shown how the developed functionality enables a number of usage scenarios where the interoperability of SMW and the wider Semantic Web is leveraged. The enabled usage scenarios include; i) Bootstrapping a non-trivial wiki structure from existing RDF data, ii) Round-tripping of semantic data between SMW and the RDF data format, for community collaboration of the data while stored in SMW, and iii) Creating mash-ups of existing, automatically imported data and manually created presentations of this data. Being able to combine the powerful querying and templating features of SMW with the increasing amounts of biomedical datasets available as RDF has enabled a new, easy to use platform for exploring and working with biomedical datasets. This was demonstrated with two case studies utilising linking data between genes and diseases as well as data from cheminformatics/metabolomics.

## Abbreviations

AJAX: Asynchronous Javascript and XML. A technology to access a web service from Javascript, for receiving content or performing actions
OWL: Web ontology language
RAM: Random-access memory
RDF: Resource description framework
SMW: Semantic MediaWiki
SPARQL: SPARQL protocol and RDF query language
URI: Uniform resource identifier
